# Evaluating Patient Participation in Clinical Trials for CLL and SLE in Germany—A Mixed-Methods Study on Enrollment Potential Based on Claims Data

**DOI:** 10.3390/healthcare12212127

**Published:** 2024-10-25

**Authors:** Ann-Sophie Kuschel, Rianne Ernst, Jan-Philipp Beck, Kathrin Borchert, Sebastian Braun, Thorsten Ruppert, Matthias Meergans

**Affiliations:** 1 Vintura GmbH, Münchner Technologiezentrum, Agnes-Pockels-Bogen 1, 80992 Munich, Germany; rernst@vintura.com (R.E.); jpbeck@vintura.com (J.-P.B.); 2 Xcenda GmbH, Lange Laube 31, 30159 Hannover, Germany; kathrin.borchert@cencora.com (K.B.); sebastian.braun@cencora.com (S.B.); 3 VFA (German Association of Research-Based Pharmaceutical Companies), 10117 Berlin, Germany; t.ruppert@vfa.de (T.R.); m.meergans@vfa.de (M.M.)

**Keywords:** Germany, pharmaceutical industry, clinical trials, healthcare, innovation, oncology, chronic diseases, statutory health insurance, InGef database

## Abstract

**Background:** In recent years, the pharmaceutical industry in Germany has faced a significant decline in the number of clinical trials conducted. **Methods:** This study evaluates patient participation in clinical trials for oncology and chronic diseases in Germany, integrating quantitative and qualitative research. Data from the Institute for Applied Health Research Berlin (InGef (Institut für angewandte Gesundheitsforschung, Berlin, Germany)), covering about 88% of the German population, and expert interviews were used. **Results:** In 2022, 84.6% of 47,305 systemic lupus erythematosus patients (SLE) and 11.9% of 102,300 chronic lymphocytic leukemia patients (CLL) received guideline-based care based on study definitions. Eligibility for clinical trials between 2017 and 2022 was estimated for 8272 SLE and 886 CLL patients, with the actual enrolment of 21 of 2221 SLE patients and 86 of 340 CLL patients reflecting the respective potential. **Conclusions:** Findings indicate an unexploited potential to enroll patients with chronic diseases compared to the relatively higher enrolment rates observed for oncology diseases, such as CLL. Securing the continuation of clinical trials and utilizing the value of trial participation is of importance for strengthening Germany as an innovation hub and for ensuring that patients have timely access to medical innovations.

## 1. Introduction

Germany has long been recognized as a pivotal player in the global pharmaceutical industry, particularly in research and development (R&D) activities. The German Association of Research-based Pharmaceutical Companies (Verband Forschender Arzneimittelhersteller e.V., vfa, Berlin, Germany) has conducted a study that underscores Germany’s significant contributions, especially evident during the COVID-19 pandemic. Despite this, the study reveals that Germany’s position as a leading location for pharmaceutical innovation is facing challenges [1].

The analysis points to a worrying trend, namely that Germany is experiencing a decline in its position as a leading location for pharmaceutical research and development, with a widening gap from the global forefront. This decline is particularly noticeable in the relative number of clinical trials (studies per inhabitants) conducted within the country, which has implications for its standing worldwide. Germany falls in the lowest category in Europe (EU) and is far behind peer countries like Spain, France, and the United Kingdom. This trend is alarming because it could lead to German patients missing out on an important access option to benefit from innovative investigational compounds many years prior to regular access, and which might affect the quality of the healthcare they receive [1].

The objective of this study was to gain a deeper understanding of the current state of clinical trial involvement in Germany in two indications (oncology vs. chronic disease) and to assess the impact of this situation on patients and the healthcare system. In order to achieve this objective, a combination of quantitative and qualitative research methods was employed.

## 2. Methods

The study makes use of data from the statutory health insurance (SHI), which insures approximately 88% of the German population, or approximately 73 million individuals [2,3]. The SHI system comprises approximately 96 independent health insurance providers [4], each offering a comprehensive benefit package as mandated by social law. This setup ensures nearly complete coverage of healthcare costs, with minimal co-payments by patients. The SHI’s payments to healthcare providers account for the majority of the total healthcare costs for individual patients. While the SHI records all reimbursed tests and procedures, it does not have access to clinical data such as lab results or disease severity scores.

The analysis is based on claims data from the Institute for Applied Health Research Berlin GmbH (InGef) research database, which contains a representative sample of approximately 4 million individuals for research purposes, mirroring the German population’s structure in terms of age, gender, and regional distribution. This sample includes data from over 50 health insurances and represents 4.7% of the German population and 5.4% of the SHI-insured population as of 2022 [3]. The InGef database is regarded as a reliable source of information regarding external validity with regard to morbidity, mortality, and medication usage patterns [5]. According to the Good Practice of Secondary Data Analysis (GPS), consultation with an ethics committee is not required for analyses based exclusively on secondary data in Germany [6].

The database monitors the insurance status of nearly 80% of the population for up to six years (2017–2022). It encompasses comprehensive information on healthcare sectors, including patient demographics, inpatients, outpatients, pharmacy services, remedies, devices, and aids, as well as data on the incapacity to work and sick leave payments.

A structured approach was employed to calculate the number of participants in clinical trials for systemic lupus erythematosus (SLE) and chronic lymphocytic leukemia (CLL) within Germany. Both diseases are distinctly classified according to the ICD-10-GM and represent the oncology and chronic disease trial situations, respectively. Furthermore, indications were selected based on the availability of clinical trials in Germany, the innovative treatment landscape to potentially reflect changes in treatment pathways, and the availability of scientific experts and patient associations in the respective indications in Germany.

The methodology employed involved adapting the research timeframe to align with the data available in the InGef database, which spanned from 2017 to 2022. The focus was on clinical trials conducted in Germany for the respective indications of SLE and CLL, specifically those that commenced and concluded patient enrolment during this period. To identify the population, the relevant inclusion and exclusion criteria from clinical trials for SLE and CLL patients conducted in Germany during the specified time period were extracted from CT.gov. These criteria were then applied to the InGef data pool of patients diagnosed with SLE or CLL from 2017 to 2022. This enabled the identification of a subset of patients who met the criteria for potential participation in the trials.

To identify SLE patients potentially eligible for clinical trials, the following inclusion criteria were applied: 1. patients with an ICD-10-GM diagnosis for SLE (M32.-) in the inpatient sector (primary or secondary discharge date diagnosis) and/or at least two different quarters (M2Q criterion) in the outpatient sector (verified diagnosis); 2. patients aged at least 18 years; 3. patients without diagnosis codes indicating pregnancy or breastfeeding; 4. patients prescribed at least one background medication (selected corticosteroids, immunosuppressants or immunomodulatory agents, anti-malarials, NSAIDs) identified by ATC or OPS codes; and 5. patients without chronic infections.

CLL patients potentially eligible for clinical trials were identified based on the following inclusion criteria: 1. patients with at least one ICD-10-GM diagnosis code for CLL (C91.1.-) in the inpatient sector (primary or secondary discharge date diagnosis) and/or at least two in different quarters (M2Q criterion) in the outpatient sector (verified diagnosis); 2. patients aged at least 18 years; 3. patients with relapsed/refractory (RR) CLL, defined as at least one prescription for RR disease recommended for follow-up treatment identified by ATC and OPS codes (selected protein kinase inhibitors, monoclonal antibodies and antibody drug conjugates, other antineoplastic agents licensed for first-line treatment); 4. patients without a diagnosis code indicating transformation of CLL; and 5. patients without malignancies other than CLL.

All results from the InGef research database were extrapolated to the German population based on the underlying analysis sample in the InGef research database and the German population according to the data of the Federal Office of Statistics (DESTATIS) for the year 2022 [2].

Furthermore, we sought to validate our calculated numbers by comparing them with data from publicly available sources. This included a comparison of the planned number of clinical trial participants listed on PharmNet.Bund with the actual patient numbers recorded in EU CTR [7,8].

Aiming for an increased robustness of our study and to ensure the validity of our findings, we integrated a qualitative research component in the form of expert interviews, employing techniques to enhance the robustness of the interviews. In order to achieve a high level of reliability, it is essential to utilize a structured format comprising a pre-defined set of open-ended questions, trained interviewers, and the implementation of consistency checks. These practices help to ensure that expert interviews yield reliable and valid data, thereby making them a robust method for qualitative research. These interviews were conducted with scientific experts and patient experts (patient advocacy groups—PAGs) with a particular focus on SLE and CLL. Additionally, interviews were conducted with experts across various medical specialties (obesity, cardiovascular diseases) to gain a broader perspective and more generalized insights.

Firstly, nine scientific experts were identified and engaged with (three in SLE, three in CLL, two in obesity, and one in cardiovascular diseases), each of whom are recognized experts in their respective fields. The selection process was based on a review of relevant stakeholders in the respective indications, who were identified as contributing to national guidelines or having a high number of publications related to the indication. A total of 110 stakeholders were invited to participate, and nine scientific experts expressed willingness to take part. In addition, we consulted with three patient advocacy groups, one representing individuals with SLE and two for cystic fibrosis. These consultations were deemed relevant due to significant changes based on innovations in the treatment environment. Despite efforts to engage with patient advocacy groups also representing CLL, no other group was participating. Furthermore, consultations were held with the clinical research organizations, and the study coordinating association in Germany and two industry experts specialized in the development of clinical trials. The objective was to obtain insights that would not only validate our quantitative data but also provide a deeper understanding of the real-world implications of our findings.

Secondly, the interviews were structured to cover a range of topics pertinent to SLE and CLL treatment and research. These included discussions on current challenges in clinical trials and the potential impact of clinical trials on standard treatment protocols. The insights from these interviews were then analyzed and synthesized.

## 3. Results

### 3.1. Results Related to Patients with SLE

In 2022, there were 47,305 prevalent patients with SLE in the InGef database extrapolated for Germany. The underlying SLE patients treated with routine care identified in the database were on average 55.4 years old (±16.3) and 84% were female. Of these, 84.6% (40,041) received routine care in accordance with German treatment guidelines (selected anti-malarials, topical glucocorticoids, systemic glucocorticoids, immunosuppressants or immunomodulatory agents, topical and oral calcineurin inhibitors, non-steroidal anti-inflammatory drugs) [9]. Another 0.9% (404 out of 47,305) received SLE-related therapies (treatments within the broader ATC cluster of routine care) that are not part of routine care. A total of 14.5% (6860 out of 47,305) of patients did not receive routine care according to German guidelines nor SLE-related therapies. The severity of SLE among patients varies, with 61% having mild, 34% having mild to moderate, 2.4% having moderate, 9.7% having moderate to severe, and 2.1% having severe conditions, when applying specific treatments identified in the EULAR recommendations for the management of SLE (double counting possible) as a proxy for disease severity [10].

The de facto use of belimumab, an approved treatment for highly active SLE, has grown steadily in prevalent SLE patients from 2018 to 2022, with a range of 3.3% to 5.6%. Standard therapies such as hydroxychloroquine and prednisolone have a usage rate of 39.5% to 46.5% and 39.7% to 44.2%, respectively.

**Figure 1:**
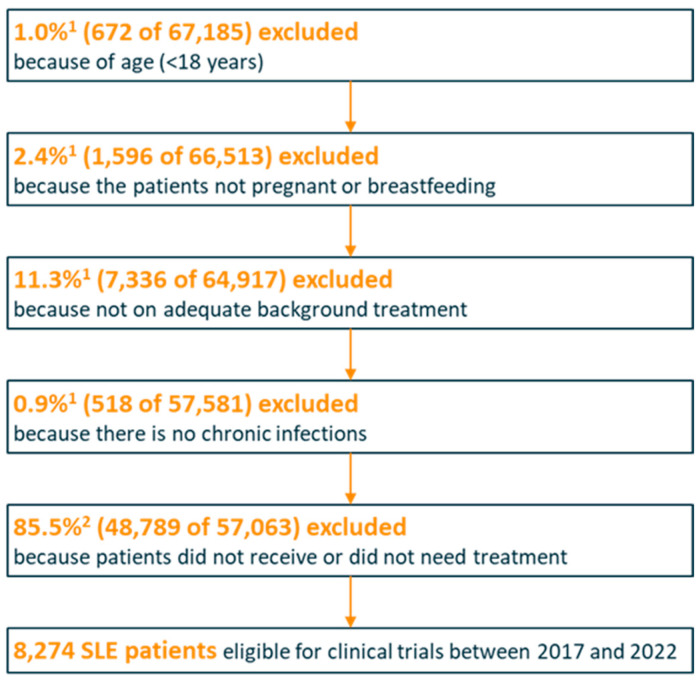
SLE patients eligible for clinical trials between 2017 and 2022. ^1^ Based on InGef data evaluation; ^2^ based on percentage of SLE patients who did not receive routine care according to German guidelines nor SLE-related therapies.

From 2017 to 2022, the extrapolated 8,272 SLE patients were estimated to be eligible for clinical trials based on a total set of 67,185 patients coded with SLE in this time period (Figure 1). Based on the recommendations of the qualitative research, a substantial percentage of 14.5% of patients might not require further medical treatment options and were therefore also excluded. These patients are eligible for clinical trials. In the same time frame, 10 clinical trials were identified in CT.gov based on the search criteria. Of the 2320 total anticipated trial patients, 121 were planned to be enrolled from Germany. Of these, 21 were actually enrolled in the 10 active clinical trials in Germany. In the case of SLE patients with routine care, approximately 20% of the total healthcare costs were related to disease-specific treatment, with pharmaceutical expenses accounting for nearly half of these costs. The average annual cost per SLE patient was approximately EUR 2200, with the majority of expenses arising from medication (approximately EUR 1000 (48%)). For SLE, the cumulative mean pharmaceutical costs invested by sponsors for patients actually enrolled in clinical trials (n = 21) between 2017 and 2022 were approximately EUR 30,000, assuming a two-year clinical trial participation.

### 3.2. Results Related to Patients with CLL

In 2022, there were 102,300 prevalent patients with CLL in the InGef database extrapolated for Germany. The characteristics of the underlying sample in the InGef database treated with routine care (n = 541) showed that patients were on average 73.2 years of age (±9.9) and 62% were female. A total of 12,129 CLL patients (11.9% of the total) received routine care in accordance with the German treatment guidelines (acalabrutinib, ibrutinib, zanubritinib, obinutuzumab, venetoclax, rituximab, idelalisib) [11,12]. Of the remaining 102,300 patients, 10,403 (10.2%) were treated with CLL-related therapies (any antineoplastic agents) that were not recommended in the current treatment guidelines. The majority of patients, 79,768 (78.0%), did not receive any CLL-related treatment.

**Figure 2:**
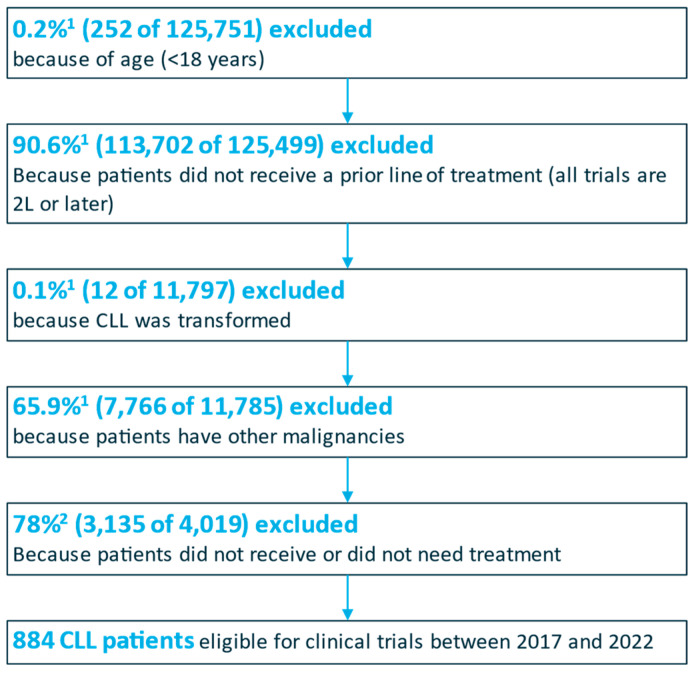
CLL patients eligible for clinical trials between 2017 and 2022. ^1^ Based on InGef data evaluation; ^2^ based on percentage of CLL patients not receiving any antineoplastic treatment according to German guidelines nor CLL-related therapies.

Regarding CLL, 886 patients were estimated to be eligible for clinical trials between 2017 and 2022 based on a total set of 125,751 patients coded with CLL in this time period (Figure 2). Following the recommendations of scientific experts, an exclusion of 78% of patients was applied on the basis of the identified number of patients not receiving any antineoplastic treatment. In the same time frame, three clinical trials were identified in CT.gov based on the search criteria. Of the 332 total anticipated trial patients, 94 were planned to be enrolled from Germany. Of these, 86 were actually enrolled. 

In the CLL patient cohort receiving routine care treatment, disease-specific treatment costs constituted approximately 80% of the total healthcare expenses, with a staggering 92% of these costs stemming from pharmaceuticals. The mean annual cost per CLL patient was about EUR 51,000, with drug expenses accounting for EUR 47,000.

In the case of CLL, the sponsors were estimated to have covered EUR 2,000,000 in cumulative medication costs for patients actually enrolled (n = 86), based on an estimated six-month clinical trial participation period.

### 3.3. Results Obtained Through Qualitative Research

According to different experts engaged through interviews, there is a significant difference in the actual enrollment for clinical trials in oncology indications versus chronic widespread diseases. The discrepancies observed can be attributed to the challenges encountered in the clinical infrastructure (with oncology being a leading sector), the recruitment and informed consent of patients, referrals by physicians, and the lengthy bureaucratic procedures for contracting, as well as the need for new therapeutic options.

## 4. Discussion

In summary, our findings indicate an unexploited potential to enroll patients with chronic diseases compared to the relatively higher enrolment rates observed for oncology diseases, such as CLL.

The majority of SLE patients (84.6%) were treated in accordance with German treatment guidelines. The discussions with SLE experts supported the number of patients receiving SLE-related treatments versus those receiving no SLE-related treatments identified in the German claims database. The experts indicated that the patients receiving no SLE-related treatment are most likely to have mild symptoms and do not require any treatment [13]. However, from a gold standard perspective, the percentage of hydroxychloroquine should be up to 80%, taking adverse events into consideration. The utilization of biologics is perceived by scientific experts to be below the number of patients requiring them, also based on the EULAR recommendations for the management of SLE [10]. An evaluation of the claims data based on the EULAR treatment recommendations also indicates a higher need for biologics, with approximately 14% of SLE patients having moderate to severe symptoms in 2022 (potentially double counting due to the treatment recommendations). Both figures indicate, according to the interviewed experts, that the prescription behavior in Germany is more reserved regarding biologics and that new therapy options might not be sufficiently adopted.

A significant proportion of patients with an ICD-10-GM code for CLL was observed to not receive any CLL-related therapy (78.0%), while 11.9% received a treatment in accordance with German guidelines and 10.2% received antineoplastic agents, although they were not up to date with current guideline recommendations. The high percentage of non-CLL treated patients is attributed by experts to the high percentage of patients with CLL being either in remission or not requiring any treatment due to a mild course of the disease. The total number of CLL-prevalent patients was perceived to be underreported due to a well-known discrepancy between reality and claims data, which is attributed to a lag in the identification and diagnosis of the disease [14,15,16]. Further limitations can be attributed to the lack of clinical data and the absence of disease and patient-related characteristics in the claims database [17]. The number of individuals in the eligible population may be subject to some uncertainty due to the inclusion of additional characteristics in clinical trials. Also, the anonymization of data is a crucial step in ensuring the privacy of individuals. However, this process inevitably restricts access to detailed person-level data, which may in turn limit the depth of certain analyses [17]. Finally, it is important to note that claims data are primarily collected for reimbursement purposes, not for epidemiological research. Consequently, the database captures only those patients who sought medical care and received a diagnosis that triggered reimbursement. Undiagnosed cases or those managed without the formal documentation of an ICD-10-GM code were not included in our analysis. Furthermore, it may overrepresent more severe cases requiring medical attention while underrepresenting milder or asymptomatic infections that did not prompt a physician visit.

The findings indicate that patients with chronic diseases, such as severe SLE, show lower enrollment rates than patients suffering from cancer, such as CLL. Hence, specifically for chronic and non-oncological conditions, there may be a greater and untapped potential for widening participation in clinical studies. A comparison of potential and actual patients eligible for clinical trials indicates that SLE patients in Germany are less likely to participate in clinical trials and the clinical infrastructure (i.e., specialized centers for rheumatology or dermatology) might be less mature than those with CLL. This suggests that there is a need to increase participation to potentially improve patient outcomes. According to experts in clinical trial design based on the qualitative evaluation, an inclusion of 10 to 20% of the total eligible population is a realistic threshold to consider in clinical trial planning. It is important to consider the number of eligible patients with caution, as an additional step of excluding patients receiving no treatment most likely due to the course of the disease (14.5%) is necessary in this funnel. This was based on expert recommendations, as these patients would not participate in clinical trials. For SLE, the planned number of patients to be included in a clinical trial was within this threshold of eligible patients. However, the actual number of patients enrolled in the clinical trial was significantly lower. According to experts, this is due to a lack of information provided to patients about potential treatment alternatives in clinical trials, as well as to community physicians and specialists.

The number of planned patients to be enrolled in the CLL clinical trial met the threshold of 10 to 20% of the eligible population. It was anticipated that almost all planned patients for Germany would be included in the clinical trial.

Expert interviews are a valuable source of in-depth insights into clinical practice. However, they are not without limitations. These include the potential for selection bias among experts and the possibility of personal biases influencing the responses of the experts. Additionally, findings may not be easily generalizable. The presence and behavior of the interviewer can also influence responses, introducing further bias. More research should be conducted to understand individual patient factors, such as patient willingness, economic conditions, and psychological or social factors. A non-life-threatening condition impacts therapeutic need and highlights the remaining hurdles in German clinical trials and the barriers to providing access to innovative treatments for patients with chronic widespread diseases. Germany has still a large untapped potential to enroll patients in clinical trials and to make innovative treatments available to patients in need. Finally, more research on the root causes is desirable in order to devise measures that could lead to patient organizations and healthcare professionals informing patients (incl. those with non-oncological chronic conditions) to be proactive in seizing the opportunity to participate in clinical research.

## 5. Conclusions

In clinical trials, the requisite number of study participants is predetermined by statistical calculations. In multinational studies, certain enrolment quotas are well established for participating countries with a competitive recruitment scheme. In the event that a country does not fulfill its committed quota, the trial initiator is obliged to attempt to compensate for this by recruiting additional participants from other countries. The lower enrollment rates for SLE compared to CLL in Germany indicate that non-oncological conditions may face challenges in clinical trial participation. Addressing these challenges is important for ensuring that patients with chronic diseases have access to innovative treatments and for maintaining Germany’s role in pharmaceutical research and development. Future efforts should aim to increase engagement from both patients and healthcare providers, improve the dissemination of information about clinical trials, and reduce administrative barriers to participation.

## Data Availability

Data is contained within the article.
